# Effects of knee osteoarthritis severity on inter-joint coordination and gait variability as measured by hip-knee cyclograms

**DOI:** 10.1038/s41598-020-80237-w

**Published:** 2021-01-19

**Authors:** Jae Hyeon Park, Hyojin Lee, Jae-sung Cho, Inyoung Kim, Jongshill Lee, Seong Ho Jang

**Affiliations:** 1 Department of Rehabilitation Medicine, Hanyang University Guri Hospital, Guri-si, Gyeonggi-do 11923 Republic of Korea; 2 Department of Biomedical Engineering, Hanyang University, Seoul, 04763 Republic of Korea; 3 Korea Orthopedics and Rehabilitation Engineering Center (KOREC), Incheon, 21417 Republic of Korea; 4 Department of Rehabilitation Medicine, Hanyang University College of Medicine, Seoul, 04763 Republic of Korea

**Keywords:** Osteoarthritis, Ageing, Outcomes research, Disability

## Abstract

Inter-joint coordination and gait variability in knee osteoarthritis (KOA) has not been well investigated. Hip-knee cyclograms can visualize the relationship between the hip and knee joint simultaneously. The aim of this study was to elucidate differences in inter-joint coordination and gait variability with respect to KOA severity using hip-knee cyclograms. Fifty participants with KOA (early KOA, n = 20; advanced KOA, n = 30) and 26 participants (≥ 50 years) without KOA were recruited. We analyzed inter-joint coordination by hip-knee cyclogram parameters including range of motion (RoM), center of mass (CoM), perimeter, and area. Gait variability was assessed by the coefficient of variance (CV) of hip-knee cyclogram parameters. Knee RoM was significantly reduced and total perimeter tended to be decreased with KOA progression. KOA patients (both early and advanced) had reduced stance phase perimeter, swing phase area, and total area than controls. Reduced knee CoM and swing phase perimeter were observed only in advanced KOA. Both KOA groups had a greater CV for CoM, knee RoM, perimeter (stance phase, swing phase and total) and swing phase area than the controls. Increased CV of hip RoM was only observed in advanced KOA. These results demonstrate that hip-knee cyclograms can provide insights into KOA patient gait.

## Introduction

Knee osteoarthritis (KOA) is a common degenerative disease involving whole joint structures including articular cartilage, synovium, ligaments, subchondral bone, and periarticular muscles^1^. KOA often results in pain, joint stiffness, postural imbalance, and walking difficulty for affected individuals^2,3^. Walking difficulty in patients with KOA is associated with higher risk of death, and mortality increases as walking ability worsens^4^. Thus, it is important to understand gait biomechanics to identify the pathophysiology of gait abnormalities in KOA patients.

To determine the gait characteristics in patients with KOA, previous studies have investigated the relationship between gait kinematics and KOA severity^5–8^. Differences in knee joint angles during the gait cycle between patients with KOA and healthy adults have been reported. Previous studies showed that gait kinematics of the hip joints could be affected by KOA^5,9^. Furthermore, gait variability, which a factor associated with falls^10,11^, could be affected by KOA^12–14^. Conventional gait analysis provides information regarding the angles of each joint separately during the gait cycle. In addition, it provides clinical and laboratory values, which provide quantitative information about gait and posture^15,16^. However, conventional gait analysis has not been widely used because it provides the results of many parameters separately and integration of these results is difficult for the assessment of multiple joints^15^. Furthermore, this method has limitations in assessing inter-joint coordination and gait variability for multiple joints simultaneously. Therefore, analytic methods are needed to integrate the kinematics of multiple joints.

Cyclograms are closed trajectories obtained by plotting the joint angle against other joints simultaneously through the entire gait cycle^17^. For example, hip-knee cyclograms can visualize the relationship between hip and knee joints during the gait cycle. The movement of one joint affects the movement of another during the gait cycle^18^. Therefore, cyclograms have an advantage over conventional gait analysis as they allow easy and intuitive recognition of the coupled movement of multiple joints ^17^. Cyclograms have been used to assess the inter-joint coordinated patterns in patients with various diseases such as stroke, hip osteoarthritis, and peroneal nerve palsy^19–21^. To our knowledge, no study has assessed the inter-joint coordination of patients with KOA using hip-knee cyclograms.

The purpose of this study was to investigate differences in (1) the inter-joint coordination between hip and knee joints and (2) gait variability with respect to structural severity of KOA by hip-knee cyclograms. We hypothesized that inter-joint coordination would be different and gait variability would be greater in patients with KOA compared with healthy older adults.

## Results

**Figure 1:**
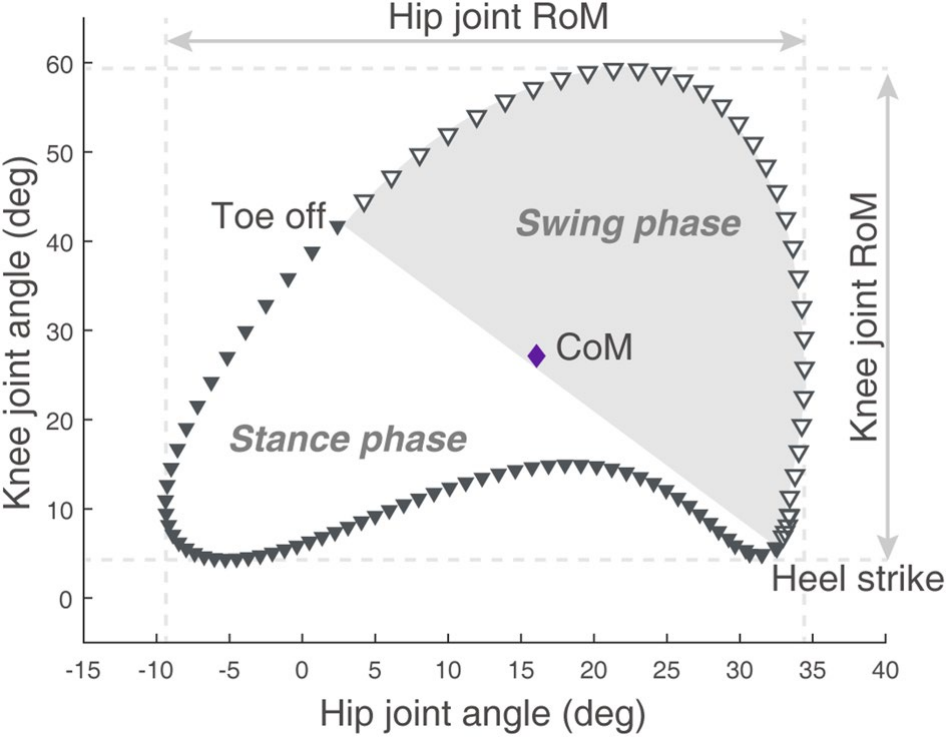
A representative sagittal plane hip-knee cyclogram. Hip joint and knee joint angles (degree) during the gait cycle are plotted in the clockwise direction on X-axis and Y-axis, respectively. Gait cycle is divided into stance phase (shown as filled inverted triangle) and swing phase (shown as open inverted triangle). *RoM* range of motion, *CoM* center of mass.

Fifty participants with KOA and 26 healthy old participants without KOA were recruited (≥ 50 years). Participants with KOA were divided based on Kellgren-Lawrence (KL) grade into early KOA (KL grade 1 and 2, n = 20) and advanced KOA (KL grade 3 and 4, n = 30). Demographic, anthropometric and clinical characteristics of the three groups are summarized in Table 1. There were no significant differences in age, height, weight, and gait speed among the three groups. Body mass index (BMI) was significantly greater in the advanced KOA group than control (p = 0.028) and early KOA groups (p = 0.019). Knee pain was observed only in KOA groups and was not significantly different between early KOA and advanced KOA groups (p = 0.415). Inter-joint coordination of kinematics represented by hip-knee cyclogram parameters including range of motion (RoM), center of mass (CoM), perimeter (stance, swing, and total) and area (stance, swing, and total) in this study are shown in Fig. 1.

**Table 1:** Demographic, anthropometric, and clinical data for the three groups.

	Control (n = 26) (Group 1)	Early KOA (n = 20) (Group 2)	Advanced KOA (n = 30) (Group 3)	*p*-value	Multiple comparisons		
					Group 1 vs. 2	Group 1 vs. 3	Group 2 vs. 3
Age (years)	61.7 ± 8.5	62.3 ± 7.2	66.1 ± 8.0	0.085	–	–	–
Sex (M:F)	8:18	5:15	8:22	0.742	–	–	–
Height (cm)	160.7 ± 7.0	158.5 ± 6.5	157.5 ± 8.3	0.264	–	–	–
Weight (kg)	61.9 ± 8.5	60.2 ± 6.5	65.4 ± 8.8	0.056	–	–	–
BMI (kg/m^2^)	23.9 ± 2.5	23.9 ± 2.4	26.5 ± 4.3	0.025*	0.689	0.028	0.019
Knee pain (VAS)	0	4.7 ± 2.6	5.2 ± 2.4	< 0.001*	< 0.001*	< 0.001*	0.415
Gait speed (m/s)	1.38 ± 0.2	1.27 ± 0.3	1.16 ± 0.3	0.186	–	–	–

Values are mean ± standard deviation or n.

*P*-value by Kruskal–Wallis test and Fisher’s exact test.

Multiple statistical differences between groups by Mann–Whitney U test with Bonferroni correction.

*KOA* knee osteoarthritis, *M* male, *F* female, *BMI* body mass index, *VAS* Visual Analogue Scale.

*Significant difference between groups.

### Hip-knee cyclogram parameters in the three groups

**Figure 2:**
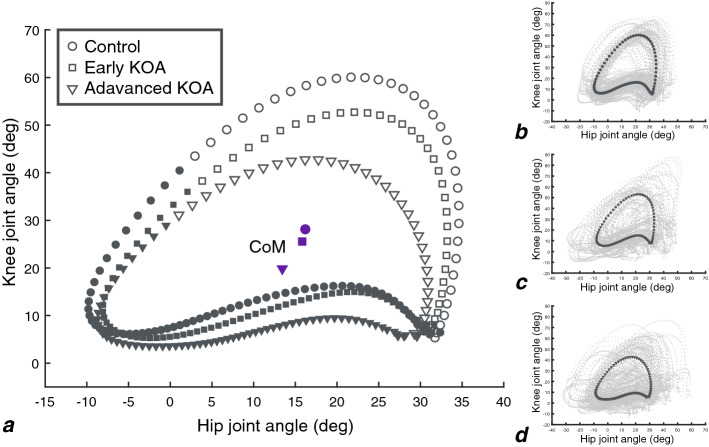
The hip-knee cyclograms of three groups (stance phase shown as filled dot, swing phase shown as empty dot). Mean hip-knee cyclograms of control, early KOA, and advanced KOA groups **(a)**. All hip knee cyclograms of control **(b)**, early KOA **(c)**, and advanced KOA **(d)**. *RoM* range of motion, *CoM* center of mass.

The average and all values for hip-knee cyclograms of the three groups are shown in Fig. 2. Differences in hip-knee cyclogram parameters are summarized in Table 2. Knee RoM was significantly decreased and total perimeter tended to be decreased with respect to KOA severity (progressive changes). Stance phase perimeter, swing phase area, and total area were significantly reduced in both early and advanced KOA groups compared with the control group, and there were no significant differences between the early and the advanced KOA groups (all KOA changes). Knee CoM and swing phase perimeter was significantly decreased in the advanced KOA group compared with the control group without a significant difference between the control and the early KOA group (only advanced changes).

**Table 2:** Hip-knee cyclogram parameters between groups.

		Control (Group 1)	Early KOA (Group 2)	Advanced KOA (Group 3)	*p*-value	Category	Multiple comparisons		
							Group 1 vs. 2	Group 1 vs. 3	Group 2 vs. 3
**RoM (deg)**									
Hip		46.69 ± 5.59	44.64 ± 4.90	44.40 ± 9.15	0.416	No difference	–	–	–
Knee		57.51 ± 8.03	51.94 ± 7.42	44.77 ± 11.49	< 0.001*	Progressive	0.010*	< 0.001*	0.013*
**CoM (deg)**									
Hip		16.19 ± 4.03	15.84 ± 8.60	13.44 ± 7.81	0.235	No difference	–	–	–
Knee		28.12 ± 5.52	25.55 ± 7.27	19.85 ± 7.92	< 0.001*	Only advanced	0.080	< 0.001*	0.029
**Perimeter (deg)**									
Stance phase		107.64 ± 15.92	92.48 ± 16.38	88.07 ± 23.00	0.001*	All KOA	0.007	< 0.001*	0.362
Swing phase		79.98 ± 11.25	81.17 ± 13.71	66.49 ± 16.15	0.001*	Only advanced	0.451	0.001*	0.001*
Total		187.62 ± 22.37	173.65 ± 18.33	154.56 ± 33.36	< 0.001*	Only advanced	0.037	< 0.001*	0.034
**Area (deg**^**2**^**)**									
Stance phase		680.89 ± 180.15	621.00 ± 163.66	590.29 ± 282.37	0.461	No difference	–	–	–
Swing phase		974.21 ± 262.18	771.35 ± 190.59	631.30 ± 303.06	< 0.001*	All KOA	0.004*	< 0.001*	0.055
Total		1655.10 ± 397.27	1392.35 ± 312.50	1221.59 ± 556.16	0.002	All KOA	0.011*	0.001*	0.191

Values are mean ± standard deviation.

*KOA* knee osteoarthritis, *CoM* center of mass, *RoM* range of motion.

*P* value by Kruskal–Wallis test and multiple statistical differences between groups by Mann–Whitney U test with Bonferroni correction.

*Significant difference between groups.

### The coefficient of variance (CV) for hip-knee cyclogram parameters in the three groups

Gait variability assessed by the CV of hip-knee cyclogram parameters are listed in Table 3. The CV of CoM, knee RoM, perimeter (stance phase, swing phase, and total) and swing phase area were significantly increased in both early and advanced KOA groups than the control group, while a significant difference between the early and the advanced KOA groups was not observed (all KOA changes). The CV of hip RoM was greater in the advanced KOA group than the control group (only advanced change).

**Table 3:** The coefficient of variance (CV) for hip-knee cyclogram parameters between groups.

		Control (Group 1)	Early KOA (Group 2)	Advanced KOA (Group 3)	*p*-value	Category	Multiple comparisons		
							Group 1 vs. 2	Group 1 vs. 3	Group 2 vs. 3
CoM		13.34 ± 7.25	61.47 ± 77.02	53.80 ± 95.23	< 0.001*	All KOA	0.001*	< 0.001*	0.707
**RoM**									
Hip		4.92 ± 2.28	6.83 ± 4.54	8.12 ± 4.50	0.038*	Only advanced	0.199	0.012*	0.251
Knee		4.36 ± 2.91	11.20 ± 8.36	11.99 ± 8.36	< 0.001*	All KOA	< 0.001*	< 0.001*	0.722
**Perimeter**									
Stance phase		4.90 ± 1.71	8.55 ± 4.19	9.60 ± 5.41	< 0.001*	All KOA	0.001*	< 0.001*	0.593
Swing phase		6.57 ± 2.84	11.28 ± 5.91	11.53 ± 7.29	0.007*	All KOA	0.002*	0.015*	0.782
Total		3.47 ± 1.31	7.29 ± 3.13	7.74 ± 5.14	< 0.001*	All KOA	< 0.001*	0.002*	0.593
**Area**									
Stance phase		15.69 ± 8.10	15.91 ± 6.73	22.66 ± 18.60	0.499	No difference	–	–	–
Swing phase		13.40 ± 8.43	24.36 ± 19.47	24.49 ± 17.39	0.004*	All KOA	0.010*	0.002*	0.678
Total		10.43 ± 5.68	15.90 ± 10.82	17.21 ± 13.32	0.047*	No difference	0.035	0.031	0.984

Values are mean ± standard deviation.

*P* value by Kruskal–Wallis test and multiple statistical differences between groups by Mann–Whitney U test with Bonferroni correction.

*KOA* knee osteoarthritis, *CoM* center of mass, *RoM* range of motion.

*Significant difference between groups.

## Discussion

In this study, we elucidated differences in the inter-joint coordination and the gait variability in response to KOA severity. Differences were observed corresponding to KOA progression (knee RoM, tendency in total perimeter), in both the early and advanced KOA groups (stance phase perimeter, swing phase area, and total area), or only in the advanced KOA group (knee CoM, swing phase perimeter). Gait variability parameters including the CV of CoM, knee RoM, perimeter (stance phase, swing phase and total) and swing phase area were greater in both early and advanced KOA groups compared with the control group. Increased CV of hip RoM was observed in only the advanced KOA group.

Consistent with our hypothesis, the results of this study demonstrated significant differences in knee RoM from the control to early KOA and from early KOA to advanced KOA. Reduced knee RoM with respect to KOA progression is in agreement with previous studies^5,7,8,22^. Changes in knee RoM between groups were more pronounced in knee flexion during the swing phase in hip-knee cyclograms. Limited knee RoM can be caused by effort to relieve pain, knee instability caused by insufficient knee extension strength, or knee flexion deformity in KOA patients^7,8^. However, hip RoM did not significantly differ among groups in this study, which may be due to the criteria used to divide patients into groups. In previous studies in which reduced hip RoM was observed in severe KOA, patients with severe KOA were defined as those who had a KL grade 4 change in knee radiography or who were indicated for joint replacement surgery^5,9^. In this study, advanced KOA was defined as a KL grade 3 or 4 in knee radiographs. This indicates that hip RoM may be affected in end-stage KOA.

Parameters of inter-joint coordination ascertained from hip-knee cyclograms also differed between groups. The total perimeter tended to be reduced with KOA progression, whereas all OA changes included stance phase perimeter, swing phase area, and total area. Additionally, swing phase perimeter and the CoM of knee demonstrated significant differences only in advanced KOA. Decreased hip-knee cyclogram parameters in KOA may result from lower knee flexion in the swing phase, which was in accordance with previous studies^5,8^. However, stance phase area showed no difference between groups, which might be attributable to increased stance phase area by decreased knee flexion during loading response in KOA. Interestingly, cyclograms showed similar trajectories for early KOA and advanced KOA in the preswing phase, and similar trajectories of control and early KOA in the terminal swing phase. These results suggested that inter-joint coordination of hip and knee joints during the stance and swing phases could be affected differently by KOA progression.

Gait variability parameters except for stance phase area and total area were increased in patients with KOA compared with healthy elderly participants, which was consistent with previous studies^12,14^. However, there was no significant difference in gait variability parameters between early KOA and advanced KOA, which might result from the characteristics of cyclograms. Cyclograms contain information about time and joint angles as parameter and variables, respectively^17^. As KOA progressed, the spatial–temporal gait variability parameters were increased and the angular gait variability parameters were decreased^12^. Therefore, there was the possibility that increased spatial–temporal gait variability and decreased angular variability might be cancelled out in advanced KOA. Furthermore, greater CV of hip RoM in advanced KOA was a notable finding, which indicated the possibility of hip joint variability affected by advanced KOA.

Information about the variability of inter-joint coordination in KOA patients is very limited, and this study is the first attempt to assess the variability of inter-joint coordination by hip-knee cyclograms. Wang TM et al. reported that the variability of inter-joint coordination derived from the continuous relative phase between KOA patients and controls did not differ significantly^23^. The continuous relative phase was calculated from the difference between phase angles of distal and proximal joints, which were obtained by the angular position and angular velocity of each joint^24^. Cyclograms were geometrically determined by joint angles^17^. These methodological differences may cause disagreement of results in the gait variability of inter-joint coordination in patients with KOA. Further research on gait variability of inter-joint coordination can help further understanding of the characteristics of gait in KOA patients.

There are several limitations in this study. First, the study population was relatively small to interpret small significant differences between the three groups and it was not possible to categorize the structural severity of KOA into more fine-grained groups. Second, it was not possible to evaluate the clinical course of gait characteristics in patients with KOA or healthy older participants due to the limitation of a cross-sectional study. Third, gait analysis was not performed with controlled walking speed, because gait analysis was not conducted on a treadmill. Gait speed was reported as an important factor affecting gait kinematics; however, we considered that it was also important to demonstrate differences in gait characteristics using a test similar to daily walking. Furthermore, the effect of gait speed on hip-knee cyclograms was reported as relatively small^20^. Fourth, only sagittal plane hip-knee cyclograms were assessed in this study. Other aspects of inter-joint coordination such as hip-knee cyclograms in other planes (transverse or frontal) or knee-ankle cyclograms were not evaluated. Therefore, further studies with a larger sample size to demonstrate the inter-joint coordination of various joints and planes at controlled walking speed are needed. Fifth, although BMI was greater in the advanced KOA group than in the control or early KOA groups, it was impossible to analyze the effect of BMI on gait in KOA patients due to the limited number of participants in this study.

In conclusion, the hip-knee cyclogram can be used as a novel parameter to demonstrate the inter-joint coordination and variability of gait in patients with KOA. Hip-knee cyclograms can visualize kinematic changes in hip and knee joints simultaneously and intuitively.

## Methods

### Participants

Fifty KOA patients and 26 asymptomatic participants (control) without KOA, who were aged 50 years and older, were recruited from four hospitals. Patients with KOA were diagnosed based on the American College of Rheumatology criteria^25^. The exclusion criteria were (1) history of intra-articular steroid injection within 6 months, (2) central or peripheral nervous system disease that could affect gait, (3) cognitive impairment, (4) contracture of lower extremity joints more than 10°, (5) dizziness or balance impairment that could affect gait, (6) history of knee surgery, and (7) lumbar disc herniation causing gait abnormality. Asymptomatic participants 50 years old or older who had no history of diagnosed KOA, knee pain, or difficulty in walking served as healthy old controls. Knee pain was assessed using the visual analogue scale (range: 0–10 cm)^26^. Knee radiographs for all patients with KOA were evaluated by a single physician (S.J.) who had more than 20 years of experience in musculoskeletal diseases. KOA patients were divided based on KL grade into early KOA (KL grade 1 and 2) and advanced KOA (KL grade 3 and 4)^27,28^. This study was approved by the Institutional Review Board of all hospitals (Institutional Review Board on Human Subjects Research and Ethics Committees Hanyang University Guri Hospital, SMG-SNU Boramae Medical Center Institutional Review Board, Ewha Womans University Mokdong Hospital Institutional Review Board and Korea University Guro Hospital Institutional Review Board). All subjects provided written informed consents before participation. All methods were performed in accordance with the present version of the Helsinki Declaration and Korean Good Clinical Guideline.

### Gait analysis

Gait analysis was performed using an Inertial Measurement Unit (IMU) sensor-based gait analysis system (Human Track, R. Biotech Co. Ltd., Seoul, Korea), which was validated in a previous study^29^. IMU sensors were placed on the foot dorsum, tibial shaft, middle of the femur, and lower abdomen. After adaptation to walking with the IMU sensors, all participants were instructed to walk a 10 m gait course at a self-selected walking speed. During the walking trials, tri-axial acceleration, angular velocity, and magnetometer values were measured at a sampling rate of 100 Hz from the seven IMU sensors. Errors in the gyroscope and accelerometers were minimized by reflecting the gain compensation value and the offset value obtained from a previous method ^30^. In addition, the alignment according to the sensor attachment position was considered while calculating the 3D joint angle^31^. Based on the calibration of the IMU, the result from the gait analysis was verified by comparing it with the result of the infrared system^29^. Trials with obstacles on the walkway, misaligned equipment, or sudden stops were excluded. The first and last strides of each gait trial were excluded from analysis.

Hip-knee cyclograms were generated by plotting sagittal plane hip (x-axis) and knee (y-axis) angles in a clockwise direction throughout the entire gait cycle (Supplementary video S1)^17^. Hip and knee joint angles for each stride were normalized to 0 to 100 points through the gait cycle. The gait cycle was divided into the stance and swing phases by the heel strike point and the toe off point, which were determined by the angular velocity of the foot dorsum. We calculated the average value for hip-knee cyclogram parameters including RoM and geometric parameters such as CoM, perimeter (stance, swing and total) and area (stance, swing and total). Gait variability was evaluated by the CV of hip knee cyclogram parameters^12^.

1 $${\mathrm{CV}}_{1\mathrm{D}}=\frac{\upsigma }{\stackrel{-}{x}}$$

2 $$\mathrm{mean }{\mathrm{CV}}_{1\mathrm{D}}=\frac{\sum_{i=1}^{N}C{V}_{1D}\left(i\right)}{N}\times 100 \left(\%\right)$$

3 $${\mathrm{CV}}_{\mathrm{x}}=\frac{{\upsigma }_{\mathrm{x}}}{\stackrel{-}{x}},\ {\mathrm{CV}}_{\mathrm{y}}=\frac{{\upsigma }_{\mathrm{y}}}{\stackrel{-}{y}}$$

4 $${\mathrm{CV}}_{2\mathrm{D}}=\sqrt{C{V}_{x}^{2}+{CV}_{y}^{2}}$$

5 $$\mathrm{mean }{\mathrm{CV}}_{2\mathrm{D}}=\frac{\sum_{i=1}^{N}C{V}_{2D}\left(i\right)}{N}\times 100 \left(\%\right)$$

In the equations, ${\mathrm{N}}$ is the number of strides, ${\mathrm{CV}}$ is the coefficient of variance, σ is the standard deviation, ${\stackrel{-}{x}}$ is the mean subscripts x, y are the x, y coordinates of the 2D features, and, subscripts 1D, 2D denote 1D features RoM, perimeter, Area, 2D features, (Co respectively

Since cyclograms consist of a set of successive data points as depicted in Fig. 1, the perimeter was calculated as the sum of the lengths of the straight lines connecting these data points. The area was the space enclosed within the perimeter.

The perimeter of the cyclogram was calculated using the following equation:

6 $${L}_{i}=\sqrt{{({\theta }_{{h}_{i}}-{\theta }_{{h}_{i+1}})}^{2}+{({\theta }_{{k}_{i}}-{\theta }_{{k}_{i+1}})}^{2}}=\Delta t\sqrt{{{\omega }_{{h}_{i}}}^{2}+{{\omega }_{{k}_{i}}}^{2}}$$

The ${\omega }_{{h}_{i}}$ and ${\omega }_{{k}_{i}}$ are the average angular rates(velocities) of the hip and the knee joints, respectively, during the time interval (∆t). The perimeter was calculated as $L=\sum_{i}{L}_{i}$, and this parameter provides information on the average joint velocity. The area surrounded by the set of successive data points was calculated by Eq. (7). The cyclogram^32^ area is representative of the conjoint range of joint movements^33^.

7 $$Area= \frac{1}{2}\sum_{i}({\theta }_{{h}_{i}}{\theta }_{{k}_{i+1}}-{\theta }_{{h}_{i+1}}{\theta }_{{k}_{i}})$$

### Statistical analysis

Participant differences between groups were compared by the Kruskal–Wallis test for continuous variables and the Fisher’s exact test for categorical variables. A *p*-value < 0.05 was considered statistically significant. Parameters that were statistically significantly different between groups were analyzed by multiple comparison using the Mann–Whitney U test with a Bonferroni-adjusted p-value (p < 0.017 [0.05/3]). Statistical analyses were performed using SPSS software version 24 (IBM/SPSS Inc., Armonk, NY, USA).

Differences in KOA severities and cyclogram parameters among the three groups were analyzed. Cyclogram parameters were categorized into three patterns as follows: progressive, all KOA, and only advanced KOA. Significant differences that were decreased or increased from the control to the early KOA group and from the early KOA to the advanced KOA group were classified as progressive. Parameters that were significantly different between both the early KOA and the advanced KOA groups with the control group were categorized as all KOA. Only advanced KOA was assigned when significant differences were observed between the advanced KOA group and the control group (or both the control and the early KOA groups).

## Supplementary Information

Supplementary Video 1.

Supplementary legend.
