# Supplementary material for: Effects of knee osteoarthritis severity on inter-joint coordination and gait variability as measured by hip-knee cyclograms
Source: Sci Rep. 2021 Jan 19;11:1789. doi: 10.1038/s41598-020-80237-w (PMC7815829; doi:10.1038/s41598-020-80237-w)
Supplement: Supplementary file 2 — Supplementary legend. [file 41598_2020_80237_MOESM2_ESM.docx]

**Effects of knee osteoarthritis severity on inter-joint coordination and gait variability as measured by hip-knee cyclograms**

**Jae Hyeon Park^1,^**^✝^**, Hyojin Lee^2,^**^✝^**, Jae-sung Cho^3^, Inyoung Kim^2^, Jongshill Lee^2,*^ & Seong Ho Jang^1,4,*^**

^1^Department of Rehabilitation Medicine, Hanyang University Guri Hospital, Gyeonggi-do 11923, Republic of Korea

^2^Department of Biomedical Engineering, Hanyang University, Seoul 04763, Korea

^3^Korea Orthopedics & Rehabilitation Engineering Center (KOREC), Incheon 21417, South Korea

^4^Department of Rehabilitation Medicine, Hanyang University College of Medicine, Seoul 04763, Korea

^✝^ These authors contributed equally to this work

* Correspondence and requests for materials should be addressed to J.S.L. (email: netlee@hanyang.ac.kr) or S.H.J. (email: [systole@hanyang.ac.kr](mailto:systole@hanyang.ac.kr))

**Supplementary Video S1.** Hip-knee cyclogram using sagittal plane hip and knee angles throughout the gait cycle (stance phase as blue dots, swing phase as red dots).
